# Cortical–Subcortical Interactions in Hypersomnia Disorders: Mechanisms Underlying Cognitive and Behavioral Aspects of the Sleep–Wake Cycle

**DOI:** 10.3389/fneur.2014.00165

**Published:** 2014-09-11

**Authors:** Linda J. Larson-Prior, Yo-El Ju, James E. Galvin

**Affiliations:** ^1^Department of Radiology, Washington University School of Medicine, St. Louis, MO, USA; ^2^Department of Neurology, Washington University School of Medicine, St. Louis, MO, USA; ^3^Departments of Neurology, New York University Langone School of Medicine, New York, NY, USA; ^4^Department of Psychiatry, New York University Langone School of Medicine, New York, NY, USA; ^5^Department of Population Health, New York University Langone School of Medicine, New York, NY, USA

**Keywords:** hypersomnia, cognitive fluctuations, sleep, review, brain networks

## Abstract

Subcortical circuits mediating sleep–wake functions have been well characterized in animal models, and corroborated by more recent human studies. Disruptions in these circuits have been identified in hypersomnia disorders (HDs) such as narcolepsy and Kleine–Levin Syndrome, as well as in neurodegenerative disorders expressing excessive daytime sleepiness. However, the behavioral expression of sleep–wake functions is not a simple on-or-off state determined by subcortical circuits, but encompasses a complex range of behaviors determined by the interaction between cortical networks and subcortical circuits. While conceived as disorders of sleep, HDs are equally disorders of wake, representing a fundamental instability in neural state characterized by lapses of alertness during wake. These episodic lapses in alertness and wakefulness are also frequently seen in neurodegenerative disorders where electroencephalogram demonstrates abnormal function in cortical regions associated with cognitive fluctuations (CFs). Moreover, functional connectivity MRI shows instability of cortical networks in individuals with CFs. We propose that the inability to stabilize neural state due to disruptions in the sleep–wake control networks is common to the sleep and cognitive dysfunctions seen in hypersomnia and neurodegenerative disorders.

## Introduction

The brain is a complex dynamic system in which interactions on multiple temporal and spatial scales enable adaptive behaviors appropriate to environmental stimuli. These interactions are accomplished not only by specific network activities that produce organismal responses to stimuli but also by the general state of the system, which is most clearly represented in the shift of state from wake to sleep. Thus, system-wide dysfunctions can occur both in the networks responsible for specific functional responses to the external world and in the less well understood networks responsible for the maintenance of and switching between neural states.

**Figure 1:**
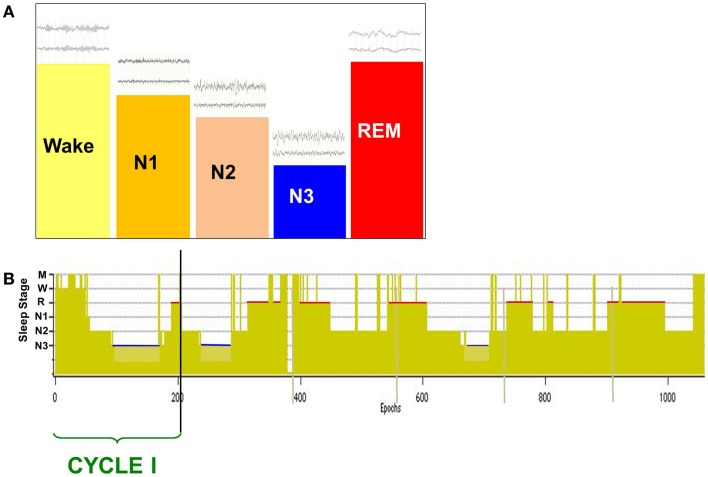
**Scalp-recorded EEG defines the neural and behavioral states of wake and sleep (A), which are charted over a full nocturnal sleep bout in a hypnogram (B)**. The descent to sleep is characterized by changes in EEG frequency content and amplitude [above state bars in **(A)**], with deep sleep (slow wave sleep, N3) characterized by large amplitude slow waves (0.5–4 Hz). Over the nocturnal sleep bout, SWS (N3) duration is reduced [blue bars in **(B)**] while REM sleep duration increases [red bars in **(B)**] and is generally longest just prior to waking. A cycle is defined by the shift from REM sleep to another sleep stage, and normal sleepers generate two to seven cycles per night.

The normal neural state transition from wake to sleep is defined by changes in scalp-recorded electroencephalogram (EEG) that exhibit a stereotypic progression through a full nocturnal sleep bout (Figure 1) (1). The transitional state from wake to sleep is characterized by a shift in EEG spectral content in which alpha (8–12 Hz) band power is reduced as theta (4–7 Hz) power increases. Behaviorally, subjects are drowsy and physically relaxed, although when questioned they do not report being asleep. Following this transitional period, as subjects descend to true sleep (N2) the scalp EEG exhibits an increase in low frequency power and characteristic spindles of sleep (7–14 Hz). Subjects then descend into slow wave sleep (SWS, N3) that is characterized by the presence of large amplitude slow (0.5–4 Hz) delta frequency waves on scalp EEG. Stages N1–N3 comprise non-rapid eye-movement (NREM) sleep and will cyclically alternate with rapid-eye-movement (2) sleep through the sleep bout. REM sleep exhibits an “active” pattern similar to that of wake, and is characterized by the distinct eye-movements from which its name was derived, peripheral atonia, and behavioral quiescence.

In 1949, Moruzzi and Magoun reported that stimulation of the brainstem reticular core produced changes in the EEG akin to those seen in arousal (3). Following the description of REM sleep by Aserinsky and Kleitman (4), studies in animal models showed the importance of the brainstem in the generation of this sleep stage (1, 5). Subsequent studies explored the neurotransmitter systems involved in the cyclic alternation of REM and NREM sleep, as well as brainstem regions active during wake, pointing to an important role for the brainstem reticular core in the control of sleep and waking. These studies clarified many mechanisms of the induction and maintenance of normal sleep, and the control of both circadian (24-h) and ultradian (90–120 min cycle of NREM/REM) rhythm generation in both animal and human subjects (1, 6–14).

Disruptions of sleep–wake and circadian cycling commonly accompany neuropsychiatric and neurodegenerative disease (15, 16). Such disruptions range from changes in the duration of nighttime sleep or specific sleep periods to disorders of circadian patterning such as seen in “sundowning” in Alzheimer’s Disease (AD) patients (15–19). In addition to overt disruption of nighttime sleep, and often considered solely a concomitant of the loss of sleep, daytime cognitive function may also be adversely impacted in these disorders. While the mechanisms by which neurocognitive and neurobehavioral dysfunction interact with sleep and circadian rhythm disruptions are currently unknown, there is clear overlap between sleep regulating regions and neurotransmitter and neural network systems affected in these disorders (15, 17, 20) that points to the potential for complex interactions between sleep and cognition.

In 1917, Constantin von Economo proposed a neurophysiological substrate for the control of the neural state transition from wake to sleep based upon the clinical and neuropathological features of a disorder in which patients exhibited abnormal sleep/wake rhythms (21, 22). This disorder, which von Eonomo termed encephalitis lethargica (EL), exhibited two subtypes with opposite effects on sleep/wake rhythms; one in which sleep duration was prolonged and intruded on waking periods and another in which patients had reduced sleep durations and difficulty in initiating or maintaining sleep (21, 23). Regardless of subtype, patients reported excessive daytime sleepiness (EDS). His observations led him to postulate the presence of an active sleep regulatory system centered in the hypothalamus (21). Von Economo’s hypothesis that the ventral hypothalamic region housed a sleep center while posterior hypothalamic regions generated the wake-state has informed studies of the neurobiology of sleep and arousal since its initial description, with later studies confirming a major role for hypothalamus in the regulation of sleep and wake (24–29).

The expression of these biological rhythms in the cortex was the focus of seminal studies by Mercia Steriade and his colleagues (30–32), who provided evidence of the role of thalamo-cortical circuits in the generation of the EEG signatures of sleep. This work was extended to show the importance of thalamus in generating EEG rhythms (33–35) while pointing out that the full expression of these rhythms required the interaction of both thalamus and cortex (30–32, 36–41). While the cortex has been suggested to play a role in the decentralized control of the homeostatic sleep drive (42–44), the current consensus puts the sleep/wake control center in subcortical circuits. However, regardless of its role in the primary control of sleep, thalamo-cortical circuitry has a clear and critical role in the regulation of cognitive and behavioral aspects of sleep and waking. Thus, the reintegration of this circuitry in theories of neural state regulation is necessary if we are to gain a true understanding of the role of sleep disregulation in pathological neural and cognitive states.

## Brief Overview of Hypersomnia Disorders

Hypersomnia disorder is an umbrella term for a group of disorders in which the primary characteristic is EDS in the face of normal or longer than normal nocturnal sleep (45). Hypersomnia disorders (HDs) are recognized as primary disorders of sleep, and it is the lack of refreshing sleep – sleep that results in a wake period in which the patient feels alert and motivated – that often drives sufferers to seek medical assistance. Yet it must be recognized that disorders of hypersomnia are equally disorders of wake, as it is the waking state in which patients report the greatest distress due to cognitive, social, or workplace dysfunction.

While most research examining neurobiological and neurophysiological substrates of these disorders have focused on the neural circuitry that produces and maintains sleep and wake, it is the interactions of these sleep-related circuits with those functioning in wake-state arousal, and how these interactions influence cognition and behavior, that must ultimately be explained if effective therapies are to be developed.

### Narcolepsy

Narcolepsy is a disorder in which sleep intrudes on daily activity while nocturnal sleep is frequently fragmented, and is classified by the ICDS-2 as a hypersomnia of central origin (46). Narcolepsy is clinically defined by a short sleep latency and two or more sleep onset REM periods (SOREMPs) during a multiple sleep latency test (MSLT) in which individuals are given four to five standardized daytime nap opportunities (46, 47). Two forms of narcolepsy are recognized; narcolepsy with cataplexy, currently named narcolepsy/hypocretin (HCRT) deficiency disorder (47), and narcolepsy without cataplexy. Narcolepsy with cataplexy is due to low levels of hypocretin-1, which can be confirmed by measurement in cerebrospinal fluid (47), and it has a strong linkage to human leukocyte antigens (48) with HLA-DBQ1*0602 mutations found in 90% of tested patients (49–51).

In 1998, two laboratories announced the discovery of a new hypothalamic peptide, one reporting its importance in feeding [orexin (ORX); (52)] and the other focused on its role in wake and sleep [HCRT; (53)]. The discovery that narcolepsy with cataplexy resulted from loss of ORX/HCRT-containing neurons in the posterior lateral hypothalamic area (pLHA) provided a fuller understanding of the symptomology of this disorder (54–56), and supported an early hypothesis put forward by von Ecomono (21). In this disorder sleep-to-wake transitions are unstable, as patients are generally unable to maintain consolidated sleep during the main nocturnal sleep period, and unable to maintain wake during the normal wake period. Individuals with narcolepsy have early onset of REM sleep; including at sleep onset, during the main sleep period and during naps, indicating a defect in the normal progression of sleep stages. Additional REM phenomena frequently associated with narcolepsy are also due to an instability between wake and REM: hallucinations in sleep–wake transitions (dreaming imagery of REM with awareness of wake), sleep paralysis (paralysis of REM sleep with awareness of wake), and REM sleep behavior disorder (dream imagery of REM sleep with muscle tonus of wake).

As with other HD, narcolepsy patients complain of memory problems and difficulties with concentration and attention (57–59). Attentional deficits, particularly in vigilance tasks, have also been reported (58, 60, 61). Attentional deficits seem to be an effect of the fluctuations of arousal that accompany this disorder, more than a deficit in attentional control in general (58, 59). These lapses of attention result in impaired vigilance over long periods that can be compensated by deploying attention in repeated shorter bouts (62).

The etiology of narcolepsy is currently unknown (63), although recent studies have provided strong experimental support for autoimmune etiology (64–67). Narcolepsy has been reported as secondary to tumors (68), head trauma (68–70), and immune-related disorders (67, 71, 72).

### Kleine–Levin syndrome

The International Classification of Sleep Disorders (ICDS-2) recognizes idiopathic and recurrent hypersomnia as distinct entities (46). The most common recurrent hypersomnia is Kleine–Levin Syndrome (KLS), a rare disorder that predominantly affects adolescent boys and is characterized by bouts of hypersomnolence during which the patient also exhibits one of the following: cognitive or mood disturbances, compulsive eating, hypersexuality, or disinhibition behaviors (47, 73). Behavioral, sleep, and mood symptoms remit in interictal periods (74).

Cognitive disturbances, unlike behavioral and mood disturbances, have been reported to outlast ictal periods (73–76). Depression and anxiety are common in this population (77, 78), and recent studies suggest that there are long-term deficits in memory and visuospatial function (73, 75, 76, 79). As these mood and cognitive symptoms are similar to those reported for idiopathic HD (iHSD), the pathophysiological mechanisms by which they are generated is expected to be similar.

Neuroimaging studies have provided some clues as to the genesis of cognitive and mood disruptions in this population. Although structural neuroimaging is generally read as normal in KLS, widespread abnormalities have been reported during ictal periods based on functional neuroimaging; with reduced blood flow to thalamus, hypothalamus, basal ganglia, and cortex (73, 80) together with hypometabolism in hypothalamus and cortex (81). Studies have shown hyperactivation in the thalamus of KLS patients during performance of a working memory task using fMRI (75, 76) that significantly differed from the activation seen in healthy control subjects and correlated with performance deficits in KLS patients.

The pathophysiology of the disorder is unknown and its diagnosis remains based upon symptomology (73, 76, 81). Interestingly, and in common with EL, a viral infection potentially associated with a subsequent autoimmune response has been proposed as a causative agent and two autopsy cases have reported inflammatory infiltrates in hypothalamus and thalamus (73, 77, 82).

### Hypersomnolence disorder

Hypersomnolence disorder (47) may include only non-refreshing sleep despite nocturnal sleep durations (idiopathic) but is more frequently coexistent with other neurological, psychological, mental, and sleep disorders (83, 84). EDS in spite of normal or long duration nocturnal sleep is characteristic of the disorder though insufficient to define it (45–47). A characteristic deterioration in waking function and general alertness is generally remarked and symptom duration must exceed 3 months with a sleep onset latency of <10 min for a clinical diagnosis to be made.

Idiopathic hypersomnia (iHSD) occurs in two forms: (1) long sleep duration, in which patients sleep in excess of 10 h per day while reporting non-refreshing sleep and EDS, and (2) normal sleep duration where sleep bouts are within normal range but EDS and non-refreshing sleep remain primary complaints. Patients report constant fatigue or a constant lack of alertness during wake periods more than sleepiness (85–87) together with great difficulty in waking after sleep. Sleep efficiency is good in these patients, and REM sleep onset latencies and durations are generally within normal range. However, changes in nocturnal sleep architecture during polysomnography (PSG) have been reported, including a reduction in the amount of SWS in the main sleep bout with normal homeostatic reduction (87–89).

In addition to non-refreshing sleep, patients report memory and attention deficits and commonly present with digestive system disorders, depression, and anxiety (84, 86). In a recent study based on self-reported cognitive changes, 79% of patients with hypersomnolence disorder (HSD) reported memory problems that included frequent forgetfulness (86). A recent study assessing sustained attention in a range of HSD patients found impaired vigilance that did not differ significantly between patient groups (90), suggesting that this is a common feature of HSD. Mood disorders are also commonly reported by individuals with iHSD, with prevalence between 15 and 25% in this population (78, 91) during asymptomatic periods. The prevalence of depressive symptoms during symptomatic episodes is greater (82), with up to 40% of patients exhibiting symptoms. The pathophysiological mechanisms for mood and cognitive symptoms in iHSD have yet to be specifically explored, although they are similar to those reported for KLS.

## Neurobiological Substrates of Sleep and Waking

### Subcortical network interactions in sleep and waking

Control of the sleep–wake cycle depends upon a widely distributed and complex neural system, many components of which have been shown to be affected by HD. The hypothalamus represents a primary control center in the regulation of this system; acting as the interface between circadian, energetic, sleep, and autonomic circuits that are all modulated in sleep (92–94). The circadian system provides information critical to the function of the sleep–wake control system, contributing one arm of the two-process model of sleep regulation (95) and interacting closely with subcortical regions to link this to the ultradian sleep–wake rhythm (96, 97). In the following, we provide a brief overview of the subcortical brain circuits involved in the regulation and control of sleep and wake together with the interactions between these systems and the cortex.

**Figure 2:**
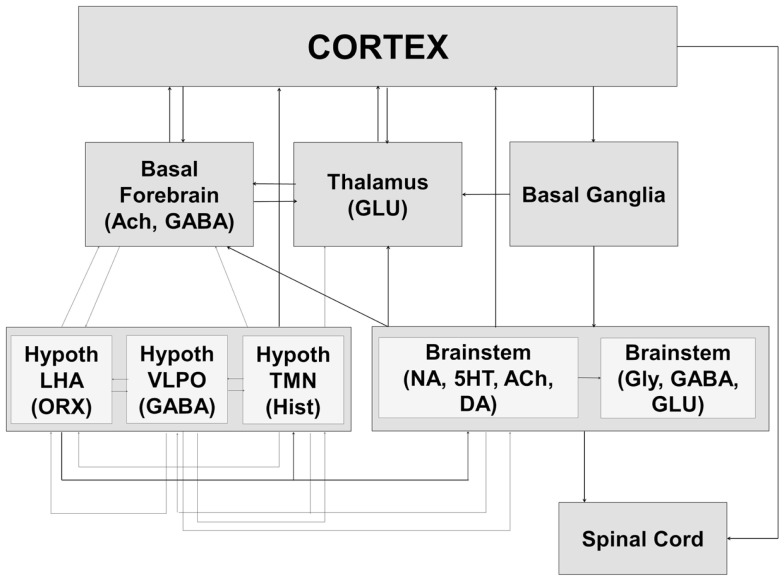
**The sleep–wake cycle is controlled by a widely distributed and complex neural system that includes interacting components of the brainstem, hypothalamus, basal ganglia, basal forebrain, and thalamus**. The interactions between these systems result in both global changes in neural state and their behavioral expression at the level of the spinal cord and brainstem (motor atonia or phasic bursts, autonomic responses) as well as the cortex (conscious awareness of external environment). Ach, acetylcholine; GABA, gamma-amino butyric acid; GLU, glutamate; Hypoth, hypothalamus; LHA, lateral hypothalamic area; ORX, orexin; VLPO, ventrolateral preoptic area; TMN, tuberomammillary nucleus; Hist, histamine; NA, noradrenaline; 5HT, serotonin; DA, dopamine; Gly, glycine.

The preoptic region of the hypothalamus is currently recognized as the major sleep-promoting brain region (27–29, 98–100), with the ventrolateral preoptic area (VLPO) and the median preoptic nucleus (MnPO) providing inhibitory drive to brain regions engaged in the induction and maintenance of the waking state (Figure 2). Recent studies have pointed to an important role for melanin concentrating hormone (MCH) neurons of the lateral hypothalamic area (LHA) in sleep-promotion (101–105). MCH neurons co-release gamma-amino butyric acid (GABA) in wake-promoting regions, thus promoting sleep (106, 107). MCH neurons are active in both NREM and REM sleep and there is evidence suggestive of a specific role in the control of REM sleep, perhaps together with GABAergic neurons found interspersed with them in the lateral hypothalamus (101, 106, 108).

**Figure 3:**
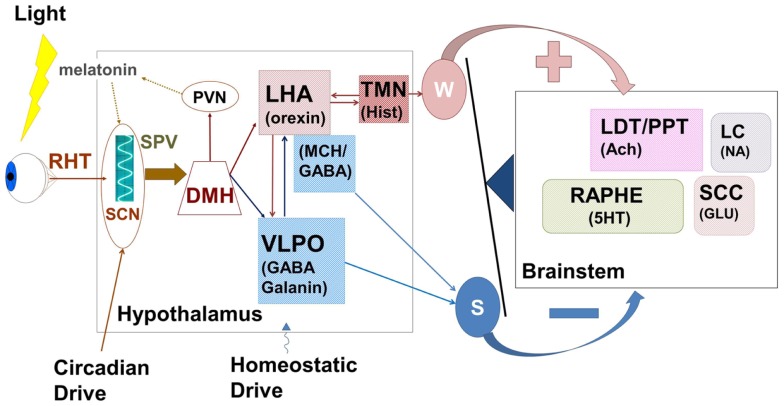
**The hypothalamus represents a central control center for the interaction between circadian rhythms paced by the suprachiasmatic nucleus (SCN) and the sleep homeostat**. Complex feedback loops within the hypothalamus, and between hypothalamus and brainstem wake-promoting centers control the putative “switch” between wake and sleep. Wake-promoting (red) signals serve to enhance activation of brainstem centers comprising the ascending reticular activating system while sleep-promoting (blue) centers inhibit this activation. In normal sleep, the state switch is rapid (<5 min) and the new state is quickly stabilized. RHT, retino-hypothalamic tract; SPV, subparaventricular zone; SCN, supra charismatic nucleus; DMH, dorsomedial hypothalamic nucleus; PVN, paraventricular hypothalamic nucleus; LHA, lateral hypothalamic area; VLPO, ventrolateral peropic area; TMN, tuberomammillary nucleus; LDT/PPT, laterodorsal tegmental/peduncular pontine tegmentum; LC, locus coeruleus; SCC, sub-coerulear complex; GABA, gamma-amino butyric acid; MCH, melanin concentrating hormone; Hist, histamine; Ach, acetylcholine; 5HT, serotonin; NA, noradrenaline; Glu, glutamate; W, wake; S, sleep.

Orexin producing cells in the LHA have been shown to play a major role in the induction and maintenance of the waking state (109–112) while linking autonomic and metabolic centers (113), thus acting as a major integrative system (Figure 3). Sleep-promoting MCH and GABA neurons are interspersed in the LHA with wake-promoting ORX neurons, providing for rapid mutual inhibition in state transitions. The waking state is generated by the inhibition of hypothalamic sleep-promoting centers together with excitation of wake-promoting centers in the hypothalamus, brainstem, and basal forebrain (Figures 2 and 3). Wake centers of the hypothalamus include both ORX cells of LHA and histaminergic (HIST) neurons of the tuberomammillary nucleus (TMN). The brainstem reticular activating system (RAS) represents the primary control system for wake (6, 26, 114–117) and consists of acetylcholine (Ach)-containing neurons in the pedunculopontine (PPT) and laterodorsal tegmental (LDT) nuclei, noradrenaline (NE)-containing cells in the locus coeruleus (LC), serotonergic (5HT) neurons of the dorsal and median raphe nuclei (RN), glutamatergic cells of the subcoeruleus complex (SCC), and dopaminergic cells of the ventral periaqueductal gray (vPAG) that are reciprocally connected to wake-promoting hypothalamic centers. Considered by some a rostral extension of the RAS, the basal forebrain includes a small population of Ach cells that are active in both wake and REM sleep, playing an important role in the generation of desynchronized electrical activity of the cortex in both states (118, 119). Basal forebrain neurons receive input from both hypothalamic and brainstem wake centers (Figure 2) and may represent a key mediating center of cortical arousal (120).

Cells of the LHA provide glutamatergic and orexinergic inputs to brainstem wake-promoting regions of the RAS and receive inhibitory drive from the VLPO. Sleep onset is initiated by activation of the sleep-promoting VLPO, which acts to inhibit both RAS and LHA. The balance between sleep-promotion and wake-promotion is accomplished by a feedback mechanism that enables the relatively rapid switch in state with a mechanism resembling an electronic switch; an analogy that led Saper and his colleagues to develop the flip–flop switch model of this state transition (26, 121). This mechanism provides for the rapid stabilization of a newly entered state, but disruption of this balance can lead to state instability where inappropriate state switches can occur.

Under normal conditions, sleep initiation moves the system into NREM sleep, following which cyclic alternations between NREM and REM sleep develop (Figure 1B) that constitute the ultradian sleep rhythm. A normal sleeper will exhibit two to seven such cycles over a nocturnal sleep bout. It is generally agreed that mesopontine brainstem nuclei contain the regulatory circuitry for the stereotypic alternations of NREM and REM sleep. Glutamatergic neurons of the SCC are proposed to be the primary inducer of REM sleep (122, 123), with current studies suggesting that hypothalamic MCH cells act to stabilize this state (106). SCC innervation of cholinergic neurons of the LDT/PPN and basal forebrain result in the desynchronized EEG characteristic of REM while inhibitory drive to wake-promoting areas such as DRN and LC aid in state stabilization. Skeletal muscle atonia is a unique characteristic of normal REM sleep and is due to SCC excitation of medullary reticular centers that act to inhibit spinal motor neurons (122–124).

In a normal nocturnal sleep bout, there is a gradual reduction in the duration of NREM sleep periods with the longest period occurring in early cycles and the late cycles frequently containing only N2 sleep. In contrast, REM sleep durations are longest in the final cycles of the nocturnal sleep bout. The reduction in N3 content over the night represents the reduction in sleep drive that is currently conceived as a homeostatic regulatory mechanism.

Sleep homeostasis is the process by which sleep propensity increases over the wake period and is dissipated during the sleep period. Early observations pointed to a strong tie between prior wake time and deep NREM sleep (125–128). The proposal of a two-process control system for the regulation of sleep and waking was put forward by Borbely and Acherman (127, 129) in a model where circadian and homeostatic processes interact to maintain and regulate sleep/wake cycling. Substantial support for this model has accrued, leading to general acceptance of its central tenets, which include the importance of slow wave activity (SWA) as a marker of both sleep need and its dissipation (126, 128, 130–134). While no central regulatory center for sleep homeostasis has been defined, a number of studies have implicated circulating neuroactive molecules as potential mediators of homeostatic control (43, 135–138).

### Thalamo-cortical network interactions in arousal and sleep

Based on a series of studies pointing to the importance of the rostral brainstem in arousal (3, 139, 140) and REM sleep (141, 142), the majority consensus among physiologists in the 1990s was that the brainstem reticular system controlled the oscillatory network responsible for wake and sleep. A large body of evidence followed these studies, reporting the importance of brainstem cholinergic systems in the behavioral and electrophysiological expression of wake and arousal. The discovery that the thalamus did not present a passive, quiescent response to the shift from wake to sleep, but instead exhibited dual processing led to the conception of sleep as a process in which the thalamus acted to “gate out” external information and thus prevent arousal (143).

A different role for the thalamus, and thalamo-cortical interaction, was provided by seminal studies investigating electrophysiological rhythms in the cortex during sleep and wake. In a series of studies investigating the role of the thalamo-cortical circuitry in sleep, Steriade and his colleagues (30–32) introduced a new, ultraslow (<1 Hz) cortical rhythm distinct from SWS that was generated in cortical neurons and projected to thalamus (32) where it served to organize the slower sleep rhythms of spindles and slow waves (30). Emphasizing its cortical origin, studies showed that thalamic lesions did not abolish the rhythm (143, 144) and that the cortex itself maintains SWA even in an isolated slice preparation (38). Further, the ultraslow rhythm is also seen in thalamic nuclei, most strongly in the reticulothalamic cells that have been shown responsible for the generation of the spindles of sleep (32). The importance of these seminal papers was in the understanding that thalamo-cortical interactions are ultimately the generators of the major sleep rhythms recorded at the scalp by which sleep states are defined. Thus, the thalamus acts as a major integrating center, generating the rhythms of both wake and sleep in concert with a widespread network encompassing brainstem, hypothalamus, and cortex. In the current understanding of sleep–wake circuitry, sleep is a state actively generated by a large and complex neural network.

In keeping with current theories of brain network function in sleep, the thalamus has been shown to continue to transmit external information to the cortex in both NREM and REM states (145–153). However, while information continues to be transmitted from the thalamus to the cortex, there are distinct differences between wake-state responses and those seen in either NREM or REM sleep. During NREM sleep, neuroimaging studies have shown that higher order cortical regions show significantly reduced or absent responses to stimuli (146, 147, 150) while responses in primary cortices appear to remain near to those noted in wake (145, 152). Further, the timing of inputs relative to thalamo-cortical waveforms characterizing NREM sleep significantly impacts the degree to which further processing occurs (150). In REM sleep, where cortical activity resembles that of wake, recent reports suggest that more complex processing may occur than seen in deep NREM sleep (149, 153) while yet remaining suppressed relative to that of wake.

While further research is required to fully describe the complex network interactions resulting in normal sleep/wake transitioning, it is increasingly clear that the hypothalamus and thalamus represent critical integration and control centers by which these states are fully expressed. As noted by von Economo and illustrated by the clear instability of state in narcolepsy and other HDs, the hypothalamus plays a critical role in the transitions between and maintenance of the states of wake and sleep. Yet, Llinas and Steriade (39) point to the thalamus as the fundamental determinant of system state, and this view is upheld by studies showing that thalamic ablation leads to a pathologically prolonged state of wake in both animals and humans (154–156). To complicate matters further, as research focuses on the role of diffusible somnogens as potential mediators of the homeostatic sleep drive (43, 135–138), some are suggesting a central role for the neocortex in the control of sleep and waking. This suggestion has received some support from recent studies showing that sleep may not be globally exhibited, but occur locally in specific cortical areas even as the organism displays behavioral wake (157–161).

**Figure 4:**
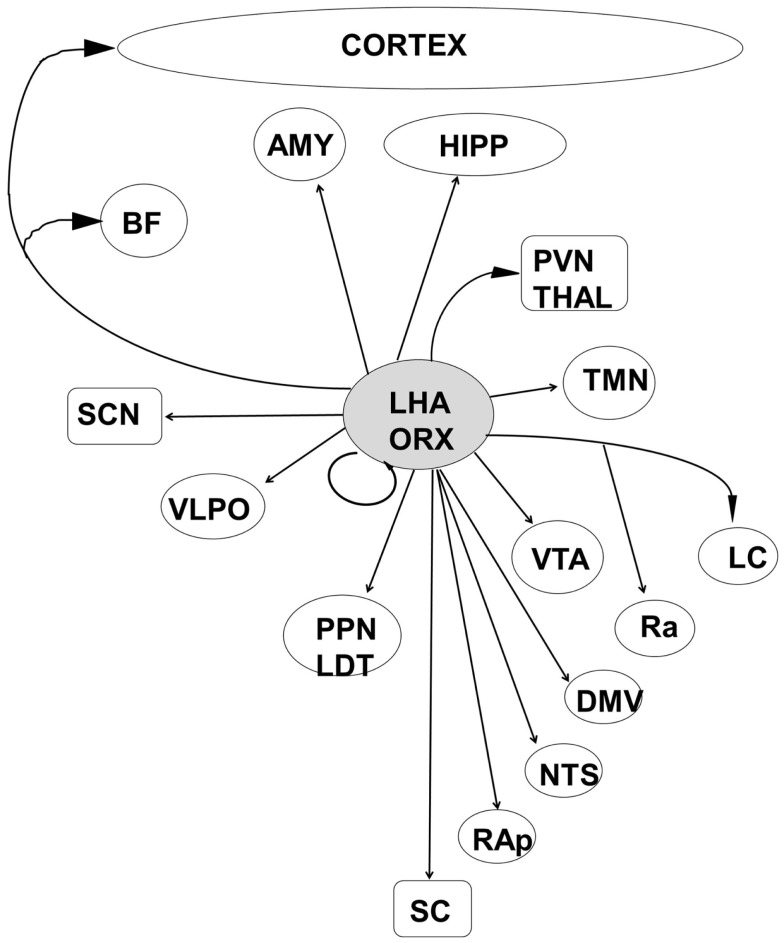
**The orexinergic cells of the posterior lateral hypothalamic area are well placed to integrate information from metabolic and autonomic centers with both sleep–wake and circadian control systems**. LHA, lateral hypothalamic area; ORX, orexin/hypocretin; HIPP, hippocampal formation; AMY, amygdala; BF, basal forebrain; PVN Thal, paraventricular nucleus of the thalamus; SCN, suprachiasmatic nucleus; VTA, ventral tegmental area; VLPO, ventrolateral preoptic area; TMN, tuberomammillary nucleus; LC, locus coeruleus; Ra, Raphe nuclei; PPN/LDT, laterodorsal tegmental/peduncular pontine tegmentum; NTS, nucleus tractus solitaries; DMV, dorsal motor nucleus of the vagus; RAp, raphe pallidus; SC, spinal cord.

**Figure 5:**
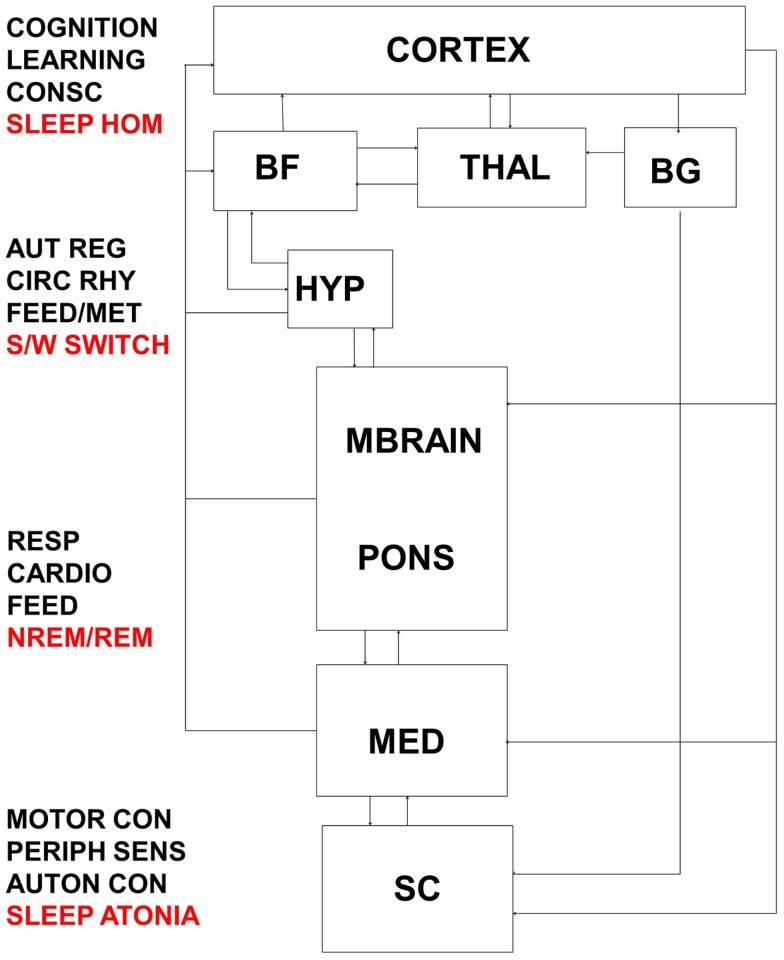
**Interactions between thalamo-cortical, hypothalamic, subcortical, brainstem, and spinal control centers are highly complex and result in the integrated response of metabolic, autonomic, and cognitive systems to daily sleep–wake rhythms**. Dysfunction at multiple levels in this system would reverberate to encompass broadly distributed symptomology. BF, basal forebrain; THAL, thalamus; BG, basal ganglia; HYP, hypothalamus; MBRAIN, midbrain; MED, medulla; SC, spinal cord; CONSC, consciousness; SLEEP HOM, sleep homeostasis; AUT REG, autonomic regulation; CIRC RHY, circadian rhythm; FEED/MET, feeding/metabolism; S/W switch, sleep/wake switch; RESP, respiratory control; CARDIO, cardiovascular control; FEED, feeding; NREM/REM, NREM/REM circuit controlling switching between sleep states; MOTOR CON, motor control; PERIPH SENS, peripheral sensation; AUTON CON, autonomic control.

Increasing evidence thus points to a widespread and highly connected network that acts in concert with the circadian rhythms generated in the suprachiasmatic nucleus of the hypothalamus to not only control the cycling of neural state between wake and sleep, but to integrate that state with metabolic and physiological systems sharing the same circadian timing (92, 96, 97). At present, recognition of the complex and redundant anatomical linkages by which the thalamo-cortical, hypothalamic, subcortical, and brainstem sleep/wake control centers interact (Figures 4 and 5) leads to a better understanding of the huge array of behavioral and physiological responses that could result from dysfunction at any level of this network.

## Cognitive Aspects of Arousal and Sleep

The complex functional integration of changes in behavioral state indexed by the alternation of wake and sleep is generally conceived as binary – the system is either in one state or the other. While changes in behavioral state may indeed show pathological binary state changes, as in the intrusion of REM sleep on wake seen in narcolepsy with cataplexy; such binary shifts in state are far less common in other HDs, where they frequently present as lapses of attention that could be considered neural states intermediate to wake and sleep.

Such intermediate states are well recognized in the attentional lapses seen with day-dreaming, boredom, or drifting off due to mild sleepiness (162, 163). Cognitive lapses have been the source of studies investigating transportation safety, where attentional lapses are a common cause of accidents (164–166). While lapses in attention are common during wake-state periods, they are exacerbated by sleep loss (167–169) where brief sleep intrusions (microsleeps) are identifiable on EEG (170).

Neuroimaging studies have pointed to neural substrates for such attentional lapses, identifying the importance of the thalamus in both the maintenance of wake and in the allocation of attentional focus under conditions of high cognitive load (168, 170). When sleep deprived, subjects showed increased thalamic activity in response to a visual attention task (168) relative to activation during rested task performance. A study looking at the neural correlates of microsleeps during a visual tracking task reported decreased thalamic activity during microsleep periods (170).

A number of studies have identified two counteracting brain networks (171–174), one most active during alert arousal [“task-positive,” (171, 173, 175, 176)] and the other dominant during periods of quiet waking or internally directed mentation [“default mode network (DMN),” (171, 177–180)]. When subjects are actively engaged in task performance, DMN activity is reduced (177, 181) while that of the “task-positive” network is enhanced (182, 183). During natural sleep (48) and under conditions of sleep deprivation (184), the anticorrelated activity noted between these two networks is reduced. While the level of anticorrelation between task-positive and DMN networks shows substantial inter-individual variability (172, 185), its maintenance reduces variability in task performance (172). In keeping with these results, brief lapses in attention during task performance have been linked to increased DMN activity (162).

Keeping in mind both the dynamic nature and the connectional complexity of the brain networks (186) controlling wake, sleep, and their intermediate states, these data suggest that dysfunction within these networks can be expressed across the full state space of the organism, resulting in diverse biobehavioral abnormalities (Figure 5). Disorders of sleep, such as the HDs, result in disordered cognitive and physiological function together with changes in the normal alternation of wake and sleep states. It is equally the case that disorders of cognition, as seen in neurodegenerative disorders such as AD and Dementia with Lewy Bodies (DLB), result in disordered sleep that includes abnormal alternations between wake and sleep states.

### Fluctuations of cognition

Cognitive fluctuations are spontaneous alterations in cognition, attention, and arousal (187) in which EDS is a prominent component and may include inappropriate sleep periods or decreased responsiveness during normal waking hours (188–190). Individuals with cognitive fluctuations (CFs) not only exhibit a higher propensity to fall asleep (hypersomnia), they also transition from a less alert to a more alert state spontaneously. Thus, the attentional lapses characteristic of CF are a manifestation of a general propensity toward inappropriate alterations in brain state.

Cognitive fluctuations are a core diagnostic criterion of DLB (188, 191), and are also seen in AD, Parkinson’s Disease (PD), and 1–3% of non-demented individuals (192–194). In DLB, CFs are more likely to be associated with daytime sleepiness, lethargy, and sleeping than in AD or vascular dementias (188, 189). Functionally, CFs result in worse clinical dementia ratings and are associated with poorer neuropsychological performance (195), greater functional impairment (193), poorer quality of life, and increased caregiver burden (196).

Diagnosis of CF generally relies upon clinical assessment (192), although caregiver reports (187, 188, 192) may also be useful. The hypothesis that fluctuations of performance on attentional tasks would reflect clinically defined and more long-term CFs has been tested in a number of studies (193, 197) with mixed results. A recent study investigating the relationship between daytime sleepiness and cognitive performance in DLB and PD patients (197), using maintenance of wake to define alertness levels, reported that CFs and level of alertness may be independent of one another, a suggestion endorsed by the study of Escandon and colleagues (195).

Electroencephalogram and neuroimaging data from several groups support the hypothesis that fluctuations reflect abnormal functional brain network interactions. Cortical slowing is a common feature of dementia, with a decrease in alpha-band amplitude in DLB accompanied by a loss of functional alpha coupling between frontal and temporal regions (198–201). While spectral abnormalities are common to a number of neurological disorders, including AD, recent studies have pointed to higher amplitude delta and theta rhythms in DLB relative to AD (199) that, together with other differences in the inter-relationships between magneto-electrical cortical rhythms may provide biomarkers of neurodegenerative disorders to aid in early diagnosis and development of therapeutics (200, 202).

Neuroimaging studies of DLB report metabolic and blood flow reductions in parietal, frontal, and occipital cortices together with gray matter atrophy that is predominant in parieto-temporal regions (203–209). Occipital lobe dysfunction identified using both emission tomography and functional magnetic resonance imaging techniques, has been associated with poor visuospatial performance and visual hallucinations in some studies (203, 207, 210). While fewer studies have focused on the brain regions or networks underlying fluctuating levels of alertness, a single photon emission tomography (SPECT) study reported an association of CFs in DLB with increased perfusion in thalamus (211), a finding partially supported by findings of hyperperfusion in both thalamus and striatum in DLB patients in whom fluctuation status was not reported (212). Additionally, regional deficits in cerebral blood flow in the precuneus and occipital lobes have been reported to differentiate DLB from AD (210, 213, 214). Using diffusion tensor imaging (DTI), loss of white matter integrity in the posterior cingulate and visual association areas has been reported in DLB (215, 216). More recent studies have focused on the role of neural network interactions, with the understanding that disparate brain regions interact to produce different brain states and activities. Increased functional connectivity between precuneus, putamen, and parietal cortex has been reported (217) with a second study reporting increases in connectivity between posterior cingulate regions and thalamus, globus pallidus, and anterior cingulate (218). Using multivariate analytic techniques in SPECT, a recent study has reported that decreased activity in bilateral parietal and parieto-temporal regions distinguished DLB from AD. While more studies are needed, particularly in regard to fluctuations of alertness, these studies point to a pattern of deficits in regional connectivity, metabolism, and blood flow that include areas important in the allocation and maintenance of attention, including the frontal cortex, parietal cortex, posterior cingulate, and precuneus. While data are mixed concerning changes in thalamic activity levels during rest, and studies addressing changes in thalamic connectivity in DLB or CFs are lacking, changes in thalamic function in patients with CF have been reported (211, 212).

## Conclusion

Over the past two decades, the central importance of sleep to both physiological and mental health has become increasingly clear. The understanding that sleep is both a local and a global phenomenon (157, 159, 160, 219) not fully constrained to the nocturnal sleep bout but locally apparent even in wake (158, 220) has provided a strong basis for the proposal that its effects on wake-state cognitive function are strong. The growing evidence of the importance of sleep to cognitive function (221–225) suggests that prolonged sleep disregulation may be a major factor in long-term cognitive decline, particularly when coupled with normal changes related to increasing age.

While the neural basis for disruptions in the normal alternation between wake and sleep state differs between HDs and the CFs seen in DLB, those changes impact the distributed and complex network controlling those states, while studies focused on CFs have yet to examine subcortical interactions that may provide evidence of such associations. The brain must maintain a balance between dynamic stability and instability; stability so as to recapitulate states and behaviors with proven efficacy, and instability so as to rapidly transition between states in response to unexpected or novel inputs. While neuroimaging studies provide important information on those brain networks involved in behavior, it is worth noting that these networks are dynamically regulated such that regional network membership shifts on millisecond timescales (186, 226–228). Further, as reported by Hellyer and colleagues (186), one property of these dynamic interactions may be the stabilization of network interactions during wake-state behaviors in which attention must be focused on behavioral tasks to provide optimal performance. Thus, we propose here that reductions in the ability to stabilize network interactions may underlie both disturbances in cognitive function that accompany sleep abnormalities and the disruptions in sleep that accompany neuropathological cognitive function.

## Conflict of Interest Statement

The authors declare that the research was conducted in the absence of any commercial or financial relationships that could be construed as a potential conflict of interest.
